# Combined Low-Frequency Ultrasound and Urokinase-Containing Microbubbles in Treatment of Femoral Artery Thrombosis in a Rabbit Model

**DOI:** 10.1371/journal.pone.0168909

**Published:** 2016-12-29

**Authors:** Yanping Zhu, Lina Guan, Yuming Mu

**Affiliations:** Department of Echocardiography, the First Affiliated Hospital of Xinjiang Medical University, Urumqi, Xinjiang, P.R. China; Monash University, AUSTRALIA

## Abstract

This paper aims to study the thrombolytic effect of low-frequency ultrasound combined with targeted urokinase-containing microbubble contrast agents on treatment of thrombosis in rabbit femoral artery; and to determine the optimal combination of parameters for achieving thrombolysis in this model. A biotinylated-avidin method was used to prepare microbubble contrast agents carrying urokinase and Arg-Gly-Asp-Ser (RGDS) peptides. Following femoral artery thrombosis in New Zealand white rabbits, microbubble contrast agents were injected intravenously, and ultrasonic exposure was applied. A 3 × 2 × 2 factorial table was applied to categorize the experimental animals based on different levels of combination of ultrasonic frequencies (Factor A: 1.6 MHz, 2.2 MHz, 2.8 MHz), doses of urokinase (Factor B: 90,000 IU/Kg, 180,000 IU/Kg) and ultrasound exposure time (Factor C: 30 min, 60 min). A total of 72 experimental animals were randomly divided into 12 groups (n = 6/group). Doppler techniques were used to assess blood flow in the distal end of the thrombotic femoral artery during the 120 minutes thrombolysis experiment. The rate of recanalization following thrombolysis was calculated, and thrombolytic efficacy was evaluated and compared. The thrombolytic recanalization rate for all experimental subjects after thrombolytic therapy was 68.1%. The optimal parameters for thrombolysis were determined to be 1) an ultrasound frequency of 2.2 MHz and 2) a 90,000 IU/kg dose of urokinase. Ultrasound exposure time (30 min vs. 60 min) had no significant effect on the thrombolytic effects. The combination of local low-frequency ultrasound radiation, targeted microbubbles, and thrombolytic urokinase induced thrombolysis of femoral artery thrombosis in a rabbit model. The ultrasonic frequency of 2.2 MHz and urokinase dose of 90,000 IU/kg induced optimal thrombolytic effects, while the application of either 30 min or 60 min of ultrasound exposure had similar effects.

## Introduction

The combined use of ultrasound, microbubble contract agent, and thrombolytic drugs has shown potential for clinical application in thrombosis treatment. Published studies have demonstrated that the thrombolytic efficacy and safety of this combination are superior to either one alone or the combination of two of them [1–5]. Thrombus-targeting microbubbles possess an affinity and high specificity for the diagnosis of a thrombus using ultrasonography, and can act synergistically with targeted thrombolysis [6, 7]. Therefore, the use of thrombus-targeting microbubbles has the potential to improve the safety and efficacy of current clinical thrombolytic therapy [8]. Acconcia et al. [9] applied 1 MHz ultrasonic irradiation to assist with the targeting of microbubble thrombolysis *in vitro*, and reported that the effects of microbubble contrast agent facilitated ultrasound thrombolysis were related to microbubble size, parameters of ultrasonic irradiation, and specific characteristics of the blood clot(s) targeted. In fact, low-frequency diagnostic ultrasonography has shown pronounced thrombolytic effects [10–15]. However, the specific factors that could potentially optimize thrombolytic efficacy *in vivo*, including selection of targeted microbubble contrast agents, ultrasound parameter and drug dosage remain unknown.

Thrombolytic drugs, including urokinase, are frequently used in the treatment of thrombotic disease. To maximize thrombolytic activity, high doses of urokinase are often used due to its dose-dependent efficacy and short half-life in the circulation. However, the use of high-dose urokinase increases the risk of bleeding in patients. An urgent need exists for more efficacious and safe pharmacotherapies, with proven thrombolytic efficacy and fewer side effects. Several studies [16–22] have found that the combination of microbubbles and ultrasonography in thrombolysis is effective, with the potential to decrease side effects and treatment costs associated with thrombolytic drugs. The present study aimed to characterize the use of targeted urokinase-containing microbubbles for the delivery of urokinase to sites of thrombosis, including the possibility of a reduction in drug dosage and clinical side effects.

Experimental studies of thrombolysis [23, 24] have reported that when the platelet glycoprotein IIb/IIIa receptor ligand (Arg-Gly-Asp-Ser, the RGDS peptide) was conjugated to microbubbles, this contrast agent showed targeted absorption to thrombi produced in *in vitro* and *in vivo* animal models. Moreover, the addition of this contrast agent enhanced the ultrasound recognition signal for the thrombosis, thereby potentially improving the diagnosis and treatment of thrombosis. In preliminary studies in our laboratory, we used the contrast agent SonoVue (Italy, BRACCO Imaging B.V.), to carry the targeting ligand RGDS and urokinase, and applied diagnostic ultrasound combined with the targeted microbubbles to treat femoral artery thrombosis in rabbits [24]. This approach of the combination of microbubbles, diagnostic ultrasound and urokinase produced the maximal thrombolytic efficacy compared with using one alone or the combination of two of them.

However, those studies did not evaluate ultrasound parameters and drug dosage. Based on previous experiments, we have known the carrier rate of Targesstar SA was 98%, which was higher than SonoVue, Since micro bubble was a vector in this study, what to be concerned is how much drug could get to the target, we used Targeson SA to carry more urokinase to the target and reduce the free drug in circulation. We used biotinylated methods to conjugate a novel, targeted microbubble (Targestar SA) with RGDS and urokinase, to create original urokinase-containing microbubbles. We then injected the microbubbles intravenously in combination with ultrasound for thrombosis therapy, and observed the subsequent thrombolytic effects of this combined therapeutic strategy. According to a factorial experimental design, the three factors affecting thrombolytic effects (ultrasound frequency and intensity, ultrasound exposure time, and thrombolytic drug dosage) were adjusted to achieve the maximally effective intravascular thrombolysis; investigate the possible mechanism(s) underlying thrombolysis; and characterize the optimal ultrasonic frequency, exposure time, and drug dosage for thrombolysis.

## Materials and Methods

### Preparation of targeting urokinase, RGDS and microbubbles

Targestar-SA (Streptavidin-coated microbubbles) was obtained from Targeson Inc. (San Diego, CA, USA) and distributed by Origin Biosciences, Nanjing, China. The outer shell of the microbubble is derivatized with streptavidin, which binds biotinylated ligands at a density of 80–220 × 10^3^ molecules per microbubble. According to the manufacturer’s instructions, the agents are suspended in aqueous saline at a concentration of approximately 1 × 10^9^ particles per mL, and have a mean diameter of approximately 2.0 μm.

Three micrograms of biotinylated urokinase (Origin Biosciences, Inc., Nanjing, China) and 3 mg of biotinylated RGDS (Origin Biosciences, Inc.) were mixed with 2 ml of the microbubble contrast agent Targestar SA (Origin Biosciences, Inc., U.S.), and gently mixed for 30 s to obtain a white, milky microbubble suspension, which stood at room temperature for 20 min before use. The preparation was evaluated for carrier rate and stability using flow cytometry. There had been flow cytometry results showed the carrier rate of Targeson SA in our one study before. The result showed that the carrier rate was greater than 98%, and rinsing the preparation with saline had no impact on the carrier rate of urokinase and RGDS. The average particle diameter of the microbubbles was 2.14 ± 1.27 μm, at a concentration of about 3.04 × 10^8^ microbubbles/ml.

### Rabbit model of femoral artery thrombosis

**Fig 1:**
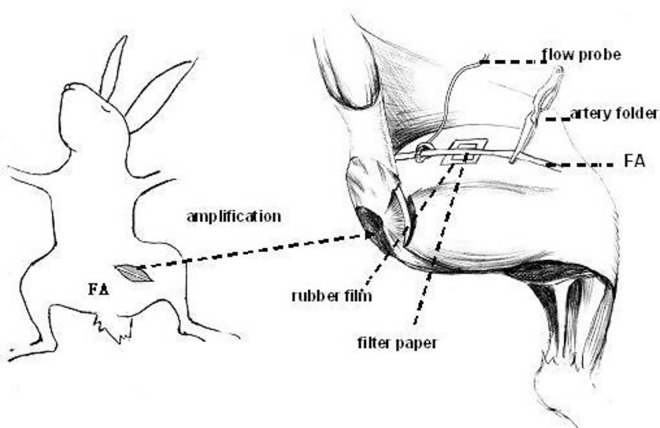
Schematic diagram of the femoral artery thrombosis model. Pulsed Doppler flowmetry was used to continuously monitor arterial blood flow. When a thrombus had formed, the pulse Doppler blood flow meter displayed values < 0.05 l/min, while the two-dimensional and color Doppler ultrasound confirmed the presence of an occlusive thrombus.

All study protocols were approved by the Ethics Committee at Xinjiang Medical University (Approval No. 20140403003). Seventy-two New Zealand white rabbits, (male and female, 1.8–2.2 kg body weight), were bred at the Animal Center of Xinjiang Medical University. Left femoral artery thrombosis was created as follows: To anesthetize the animals, sodium pentobarbital (30–40 mg/kg) was administered via ear vein with animals in the supine position. Following hair removal and skin preparation in the left groin, blunt dissection was performed to visualize the femoral artery in the left lower limb, and its corresponding superficial and deep branches were ligated. A 2.5 cm × 2.5 cm rubber film was placed in the rear wall of the artery to protect the surrounding tissues. A pulsed Doppler flowmetry probe (Transonic, TS420) was placed in the proximal femoral artery to monitor the arterial blood flow. A piece of filter paper (0.5 cm × 0.5 cm) dipped in 15% ferric chloride solution was placed between the rubber film and the posterior wall of the femoral artery. The paper was wrapped around the femoral artery, with the contact surface representing approximately 3/4 of the femoral artery circumference. After blood flow in the femoral artery was determined present, the distal end of the femoral artery was temporarily occluded with arterial clips. The clips were removed after 7–8 min and the filter paper was removed after another 20 min. Local tissues were bathed with saline solution. The details of this model of femoral artery thrombosis are illustrated in Fig 1.

### Experimental study protocol

#### Treatment groups

A 3 × 2 × 2 factorial table was used to classify the subjects into experimental groups based on the various levels of ultrasonic frequencies evaluated (Factor A: 1.6 MHz, 2.2 MHz, 2.8 MHz), doses of urokinase (Factor B: 90,000 IU/kg vs. 180,000 IU/kg) and ultrasound exposure time (Factor C: 30 min vs. 60 min). A total of 72 experimental animals were randomly divided in 12 groups, (n = 6/group) (Table 1).

**Table 1:** Classification of experimental groups based on three factors (n = 72).

Groups	The parameters of thrombolysis
A1B1C1	Ultrasound frequency of 1.6 MHz + 30 min of ultrasound exposure + targeted microbubbles loaded with 90,000 IU/Kg Urokinase
A1B1C2	Ultrasound frequency of 1.6 MHz + 30 min of ultrasound exposure + targeted microbubbles loaded with 180,000 IU/Kg Urokinase
A1B2C1	Ultrasound frequency of 1.6 MHz + 60 min of ultrasound exposure + targeted microbubbles loaded with 90,000 IU/Kg Urokinase
A1B2C2	Ultrasound frequency of 1.6 MHz + 60 min of ultrasound exposure + targeted microbubbles loaded with 180,000 IU/Kg Urokinase
A2B1C1	Ultrasound frequency of 2.2 MHz + 30 min of ultrasound exposure + targeted microbubbles loaded with 90,000 IU/Kg Urokinase
A2B1C2	Ultrasound frequency of 2.2 MHz + 30 min of ultrasound exposure + targeted microbubbles loaded with 180,000 IU/Kg Urokinase
A2B2C1	Ultrasound frequency of 2.2 MHz + 60 min of ultrasound exposure + targeted microbubbles loaded with 90,000 IU/Kg Urokinase
A2B2C2	Ultrasound frequency of 2.2 MHz + 60 min of ultrasound exposure + targeted microbubbles loaded with 180,000 IU/Kg Urokinase
A3B1C1	Ultrasound frequency of 2.8 MHz + 30 min of ultrasound exposure + targeted microbubbles loaded with 90,000 IU/Kg Urokinase
A3B1C2	Ultrasound frequency of 2.8 MHz + 30 min of ultrasound exposure + targeted microbubbles loaded with 180,000 IU/Kg Urokinase
A3B2C1	Ultrasound frequency of 2.8 MHz + 60 min of ultrasound exposure + targeted microbubbles loaded with 90,000 IU/Kg Urokinase
A3B2C2	Ultrasound frequency of 2.8 MHz + 60 min of ultrasound exposure + targeted microbubbles loaded with 180,000 IU/Kg Urokinase

#### Thrombolytic treatment

Ultrasound microbubbles conjugated with biotinylated urokinase and biotinylated RGDS, as described above, were washed up to clear the free drug before injection, and then were injected into the ear vein of each animal after thrombosis formation. One-half of the microbubble suspension was administered via intravenous bolus injection over 2 min and the remaining half was infused over 20 min through the marginal ear vein. The infusion tube was washed with 1 ml saline. After formation of the mixed thrombus, 1000 U/kg of heparin was used for anti-coagulation as part of the thrombolysis treatment.

During the course of treatment, a color Doppler ultrasonic diagnostic device (Yum Mylab 90) was used to record femoral blood flow. The ultrasound probe (LA240) was placed at the site of thrombus formation for about 10 min destruction until no contrast agents were visualized in the femoral artery. The ultrasonic frequency was set to the range of 1–4 MHz on the machine, and 1.6 MHz vs. 2.2 MHz vs. 2.8 MHz was used to irradiate the thrombus for 30 min or 60 min (according to experimental protocol). Images were acquired during a two-hour observation period.

The overall thrombolytic treatment lasted for a total of 120 minutes, whereupon the experiment was terminated and blood vessels specimens where the thrombi were located were obtained for histopathological staining (hematoxylin-eosin; H&E) to evaluate changes in the structure of blood clots during vascular thrombosis.

### Ultrasound techniques

A Yum Mylab90 color Doppler ultrasonic diagnostic device with a LA523 probe set to a frequency of 4–13 MHz and a random configuration QAS and X-STRAIN analysis software were used for visualization and synchronous recording of electrocardiogram. The long axis view of the left femoral artery was obtained through the two-dimensional ultrasound probe when the anterior and posterior walls of the intima were clearly visualized, and the probe was located at the proximal end 1.0 cm from the femoral artery bifurcation (away from the thrombus area).

### Histopathological studies

After 120 min of thrombolysis experiment, the proximal and distal vessels of the exposed part of femoral artery were ligated, and a portion of the femoral artery including the thrombosis model section was cut out. After removal of the blood vessels, animals were sacrificed by anesthetic overdose (sodium pentobarbital, 100–300 mg/kg). The specimens were fixed in 10% formalin solution and processed using conventional dehydration and paraffin embedding for sectioning and hematoxylin-eosin staining.

#### Evaluation of thrombolytic effect

Thrombosis and thrombolysis were assessed by continuously monitoring vessel blood flow using pulsed Doppler flowmetry, and the data were analyzed by use of a professional data analysis system (Power Lab system, AD Instruments Pty Ltd). The changes of femoral arterial blood flow downstream from the thrombus, before and after thrombolysis, were used to calculate the rate of recanalization after thrombolysis and evaluate thrombolytic efficacy. The vascular recanalization rate after 120 minutes of thrombolysis was calculated as: Blood flow after 120 minutes of thrombolysis/baseline arterial blood flow in the femoral artery × 100%. The method of Yamashita et al. [25] was used for analysis, resulting in the creation of 4 groups: 1) recanalization rate < 15% of baseline flow = unrealized recanalization; 2) recanalization rate of 15% to 49% of baseline blood flow; 3) recanalization rate of 50 to 75% of baseline blood flow; 4) recanalization rate > 75% of baseline blood flow. The return of femoral blood flow to 75% or greater was defined as “complete” recanalization. Two-dimensional and color Doppler ultrasound were also used for thrombolysis evaluation.

### Statistical analysis

A JMP10.0 statistical analysis software package was used for analysis. Data are expressed as mean ± standard deviation. Factorial design analysis of variance (ANOVA) was used to analyze the difference between blood flow values under various combinations. The related thrombolytic factors after 120 min of thrombolysis were analyzed by use of logistic regression, with α = 0.05 considered statistically significant.

### Outcome

#### Optimization of the parameters for thrombolysis

We applied Doppler flowmetry for dynamic detection of changes in femoral artery blood flow during thrombolytic treatment. Our results indicate that the thrombolysis recanalization rate for all animals was 68.1%, and the average time to dissolve blood clots was 50 min (Table 2). The thrombolytic effects in Groups A2B1C1 and A3B1C1 were found to be optimal, with thrombolytic effects approaching baseline blood flow of 0.143 ± 0.083 L/min and 0.164 ± 0.086 L/min, respectively, after 120 min thrombolytic treatment.

**Table 2:** Arterial blood flow monitored by Doppler flow during thrombolysis (L/min) (n = 72).

Groups	Base line	Thrombus formation	Post treatment			
			30 min	60 min	90 min	120 min
A1B1C1	0.223 ± 0.037	0.019 ± 0.003	0.028 ± 0.010	0.033 ± 0.015	0.056 ± 0.050	0.056 ± 0.049
A1B1C2	0.219 ± 0.052	0.024 ± 0.015	0.035 ± 0.038	0.042 ± 0.037	0.034 ± 0.022	0.034 ± 0.023
A1B2C1	0.208 ± 0.039	0.013 ± 0.011	0.072 ± 0.078	0.077 ± 0.075	0.112 ± 0.127	0.132 ± 0.143
A1B2C2	0.231 ± 0.055	0.017 ± 0.007	0.012 ± 0.001	0.016 ± 0.004	0.015 ± 0.004	0.022 ± 0.017
A2B1C1	0.160 ± 0.070	0.023 ± 0.016	0.108 ± 0.122	0.076 ± 0.073	0.120 ± 0.067	0.143 ± 0.083*
A2B1C2	0.250 ± 0.051	0.019 ± 0.005	0.023 ± 0.015	0.048 ± 0.045	0.027 ± 0.014	0.028 ± 0.019
A2B2C1	0.201 ± 0.043	0.028 ± 0.010	0.050 ± 0.047	0.139 ± 0.142	0.112 ± 0.120	0.120 ± 0.108
A2B2C2	0.232 ± 0.084	0.012 ± 0.008	0.013 ± 0.001	0.014 ± 0.002	0.015 ± 0.003	0.110 ± 0.081
A3B1C1	0.171 ± 0.040	0.015 ± 0.005	0.068 ± 0.067	0.105 ± 0.092	0.126 ± 0.095	0.164 ± 0.086*
A3B1C2	0.187 ± 0.060	0.015 ± 0.012	0.047 ± 0.035	0.044 ± 0.039	0.051 ± 0.040	0.070 ± 0.055
A3B2C1	0.157 ± 0.006	0.023 ± 0.006	0.050 ± 0.032	0.088 ± 0.075	0.100 ± 0.096	0.087 ± 0.064
A3B2C2	0.164 ± 0.020	0.010 ± 0.004	0.018 ± 0.004	0.018 ± 0.005	0.068 ± 0.053	0.064 ± 0.054

**Note:** The base line in Table 1 refers to the femoral artery blood flow at the start of the test, without any intervention, followed by blood flow after thrombosis and after 30 min, 60 min, 90 min, 120 min of thrombolytic therapy.

Table 3 shows the statistical significance of the relationship between ultrasonic frequency (F value = 7.58, P < 0.05) and urokinase dose (F value = 13.96, P < 0.05), and thrombolytic efficacy. No significant effect of ultrasonic irradiation time on the thrombolytic effect was observed. Also, the interactions between ultrasonic frequency and urokinase, between ultrasonic irradiation time and urokinase, and between ultrasonic frequency and ultrasonic irradiation time did not significantly affect thrombolysis. The interactions of all three factors (ultrasonic frequency, ultrasonic irradiation time, and urokinase) also failed to significantly affect thrombolysis.

**Table 3:** ANOVA analysis of femoral artery blood flow at different time point after thrombolysis compared to the baseline blood flow.

Time points	30 min		60 min		90 min		120 min	
	*F* Value	*P* value	*F* value	*P* value	*F* value	*P* value	*F* value	*P* value
Ultrasound frequency	6.24	0.00	7.19	0.00	7.48	0.00	7.58	0.00
Radiation time of ultrasound	1.77	0.19	0.12	0.73	0.08	0.78	0.00	0.98
Urokinase	10.39	0.00	15.13	0.00	17.57	0.00	13.96	0.00
Ultrasound frequency × urokinase	3.96	0.02	4.04	0.02	2.90	0.00	1.12	0.33
Radiation time of ultrasound × urokinase	0.45	0.51	0.47	0.50	0.28	0.60	2.18	0.14
Ultrasound frequency × ultrasound radiation time	0.47	0.63	0.12	0.89	0.25	0.78	0.32	0.73
Ultrasound frequency × ultrasound radiation time × urokinase	1.51	0.23	0.11	0.89	0.59	0.56	2.20	0.12

The recanalization rates in animals receiving thrombolytic therapy are shown in Table 4. The average recanalization rate was 68.1% by the end of experiment (after 120 min of thrombolysis treatment), The most favorable thrombolytic effects were seen in the groups of A2B1C1 and A3B1C1, with recanalization rates of 93% and 88.1%, respectively, which were considered as fully re-canalized rates (>75%). Partial recanalization was seen in the groups A1B2C1, A2B2C1, A2B2C2, A3B1C2, A3B2C1, and A3B2C2, with their recanalization rates in the range of 50% to 75%.

**Table 4:** The recanalization rate of femoral artery after thrombolytic therapy at various time points (%) (n = 72).

Groups	30 min	60 min	90 min	120 min	Recanalization rate
A1B1C1	12.6	14.8	25.1	25.1	15–49
A1B1C2	16.0	17.9	19.2	15.5	15–49
A1B2C1	25.3	29.4	52.6	60.5	50–75
A1B2C2	5.90	8.20	7.50	11.2	0–15
A2B1C1	84.8	59.8	94.6	93.0	75–100
A2B1C2	8.70	18.1	9.0	9.1	0–15
A2B2C1	24.8	69.1	55.6	59.6	50–75
A2B2C2	7.7	8.1	9.2	62.5	50–75
A3B1C1	49.3	82.9	84.0	88.1	75–100
A3B1C2	27.8	23.5	28.0	40.0	15–49
A3B2C1	32.6	57.0	64.2	56.6	50–75
A3B2C2	11.0	11.1	41.1	38.7	15–49
Total	–	–	–	68.1	50–75

Logistic regression screening of factors affecting thrombolytic effects after 120 min of thrombolytic therapy (Table 5) showed that the thrombolytic effects of 2.2 MHz ultrasound were 14.2× greater than that of 1.6 MHz (95% confidence interval 2.74–116.00). Moreover, the thrombolytic effects of 2.8 MHz ultrasound were 7.09× greater than that of 1.6 MHz (95% CI 1.36–55.91). The thrombolytic effect of 180,000 IU /kg urokinase was found to be 1/10th that of 1.5 mg/kg (95% confidence interval 0.02–0.36), suggesting that application of an ultrasound frequency of 2.2 MHz with 90,000 IU/kg urokinase is optimal for maximal thrombolytic efficacy. Ultrasonic irradiation time did not have a significant effect on thrombolysis.

**Table 5:** Logistic regression analysis of recanalization after 120 min thrombolytic therapy.

Parameters		*P*	*OR*	*OR* 95% CI	
				Lower limits	Upper limits
Ultrasound Frequency	2.2 MHZ/1.6 MHZ	0.0010	14.22	2.74	116.00
	2.8 MHZ/1.6 MHZ	0.0186	7.09	1.36	55.91
	2.8 MHZ/2.2 MHZ	0.3087	0.50	0.12	1.89
Urokinase	180,000 IU/Kg/90,000 IU/Kg	0.0002	0.10	0.02	0.36

#### Histopathological examination

**Fig 2:**
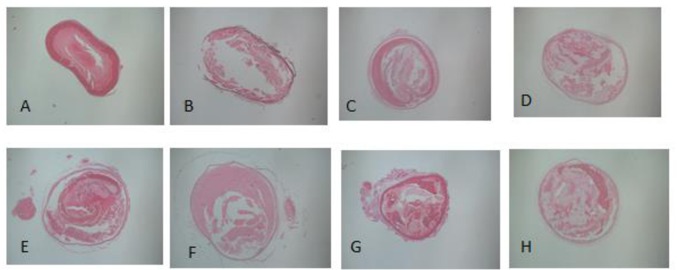
Pathological changes following femoral artery thrombosis. (A. Femoral artery thrombosis without thrombolytic therapy (observation of thrombus morphology in femoral artery thrombosis). B, C. A completely dissolved femoral artery thrombus after thrombolytic therapy. D, E, F. A partially dissolved femoral artery thrombus after thrombolytic therapy. G, H. An undissolved femoral artery thrombus after thrombolytic therapy.)

Histopathological features of the femoral artery thromboses are illustrated in Fig 2.

Under light microscopy, a mixed thrombus with platelet and fibrin was found in the endovascular lumen, with platelet trabecular formation (S1 Fig). The vascular lumen, with completely dissolved thrombus, had unclear thrombus-vascular borders with liquefied platelets and a lumen recanalization rate > 75%. Only a small number of red blood cells and inflammatory cell were found adhered to endothelial cells (S2 and S3 Figs.). Mixed thrombus was present in the partially dissolved vascular cavity, with a cavity at the edge or center of the thrombus. Scattered granular platelet trabecula were observed (S4–S6 Figs.). The vessels that were not re-canalized had numerous endovascular red cells, scattered inflammatory cells, and mixed thrombi occupying the lumen, and a small portion of dissolved granular platelets were present with lumen recanalization rates of 15%–50% (S7 and S8 Figs.). The typical pathological changes following femoral artery thrombosis were displayed in the supporting information files (S1–S8 Figs). All pathological sections showed different degrees of endothelial damage, including detachment of endothelial cells and loss of endothelial cell polarization, shedding cytoplasm, as well as thinning and fracture of the basement membrane.

## Discussion

The preparation of targeting microbubble contrast agents is the first step towards successful targeting thrombolysis using ultrasound techniques. In our preliminary studies, we applied direct conjugation of urokinase and the targeting ligand RGFDS with microbubbles, and validated the ability of these microbubbles to directly target and lyse thrombi in experimental models of femoral artery thrombosis. That study was focused on comparing the thrombolytic effect of loaded microbubbles, ultrasound treatment, and a combination of these two. We concluded that the combined use of ultrasound, microbubble, and urokinase had maximal thrombolytic efficacy. In this study, we used biotin-avidin methods to simultaneously conjugate urokinase and RGDS with microbubbles; and flow cytometry experiments revealed a conjugation rate of 98% with the Targestar SA microbubbles. When compared with the direct conjugation method, which results in a conjugation rate of less than 80%, the biotin-avidin-containing microbubbles conjugated with drug or via ligand binding were more stable. The prepared microbubble contrast agents were intravenously injected *in vivo* to selectively target femoral artery thrombi in our animal model. Our results suggest that the overall effective rate in all animals was 68.1%, with the time required to completely dissolve all blood clots averaging 50 min. Light microscopy studies showed that after thrombolysis, the thrombus fiber grids were destroyed, which documented the presence of a completely or partially dissolved thrombus. These results confirm the results of previous studies suggesting that ultrasound combined with thrombolytic drugs administered via targeted microbubbles is a novel and effective thrombolytic treatment strategy [26–30].

In this study, the frequency of ultrasonic irradiation was selected using a range of standard diagnostic ultrasound frequency, including 1.6, 2.2 and 2.8 MHz. We found that Groups A2B1C1 and A3B1C1 showed the most favorable thrombolytic effects with revascularization rates of 93% and 88.1%, suggestive of complete recanalization, with ultrasonic frequencies of 2.2 and 2.8 MHz, irradiation time of 30 min, and a urokinase dose of 90,000 IU/kg, thus demonstrating the most effective thrombolytic effect. Logistic regression analysis of data regarding thrombolytic effects after 120 min of thrombolysis showed that the ultrasound frequency of 2.2 MHz was 14.2× more effective at thrombolysis than was 1.6 MHz, and that 2.8 MHz was 7.09× more effective at thrombolysis than was 1.6 MHz. The thrombolytic effects of 180,000 IU/kg urokinase administration was 1/10 that of 90,000 IU/kg. Ultrasonic irradiation time did not significantly affect thrombolytic efficacy. Taken together, these data suggest that an ultrasound frequency of 2.2 MHz combined with low-dose urokinase represents the most advantageous parameters for thrombolytic therapy in our experimental studies.

Previous studies have conventionally employed a 30 min or 60 min duration of ultrasonic irradiation time for thrombolytic therapy [31, 32]. In the present study, we compared these two irradiation times and found no statistically significant effect of either on efficacy of thrombolytic therapy. In order to avoid the increased risk associated with longer ultrasonic irradiation, the application of 30 min ultrasonic irradiation for thrombolysis proved adequate and suggests that longer periods of irradiation will not be required in clinical practice.

Previous studies have shown that low-frequency ultrasound has shown pronounced thrombolytic effects [10–15]. It can cause a series of structural changes in thrombi via cavitation, mechanical properties, and thermal properties et al. The ability of low-frequency ultrasound to enhance the thrombolytic effects of drugs by increasing thrombus permeability and improving blood clot contact may play a role in the ability of this technique to augment thrombolysis. Our results demonstrating that an ultrasonic frequency of 2.2 MHz produces the most optimal recanalization rate may be due, in part, to a similarity in ultrasound frequency to the vibration frequency of the targeting microbubbles, thereby causing a resonance of the microbubbles to produce optimal thrombolysis.

Tissue plasminogen activator (tPA), as the first United State FDA approved cerebral infarction treatment, can be given intravenously in thrombolytic therapy within 4.5 h after the onset of stroke symptoms [33], however it is not widely used for patients in China. As a cheaper thrombolytic drug, although less specific, Urokinase has been commonly used in Chinese hospitals [34]. Since studies concerning pharmacologic thrombolytic therapy have reported that the thrombolytic efficacy of urokinase is dose-related, high doses of urokinase have been employed in the clinical setting, which results in increased risk of bleeding and consequently limits its clinical application. When the thrombus is released via degradation of its internal structure, urokinase can more easily enter the interior of thrombosis and accelerate the degradation of fibrin, thereby producing more effective thrombolysis than urokinase or ultrasound microbubbles alone. Jonathan et al. [35] studied the acoustic parameters for microbubble-assisted ultrasound thrombolysis and established an *in vitro* model using micro-clots to evaluate irradiation and microbubble ultrasound thrombolysis. Using this model, optimal acoustic parameters and microbubble properties can be identified and therefore may achieve thrombolysis without the use of thrombolytic drugs. Our results suggest that increasing the dose of thrombolytic drugs (urokinase) had little effect on recanalization. We did not find improved thrombolytic efficacy, even after doubling the urokinase dose within the targeted ultrasound microbubble. Taken together, these results argue against using higher doses of thrombolytic drugs. The biggest concern about this medication is a bleeding tendency. In this study, no bleeding was seen in the rabbits’ skin, mucous membranes, gastrointestinal tract, urinary tract, or mouth and gums.

Our study has certain limitations. First, it was limited to treatment of newly created arterial thrombi, and therapy was initiated immediately after confirmation of femoral artery thrombosis. Such circumstances are not typical of the clinical expression of arterial thrombosis. Second, the selection of the ultrasonic frequency was limited to frequencies between 1.6 and 2.8 MHz, due to the limitations associated with our instrumentation, so the effects of frequencies outside this range were not evaluated. Third, thrombosis was achieved through the use of topical ferric chloride solution and a temporary occlusion of the femoral artery, which may damage the blood vessels of the outer membrane. Consequently, whether ultrasonic irradiation may damage the adventitia remains unclear. Fourth, the use of the microbubble contrast agent Targestar SA is currently limited to experimental studies and not yet available for clinical evaluation, so the clinical applicability of our findings is untested. Lastly, the linear Arg-Gly-Asp (RGD) peptides are not specific for Glycoprotein IIb/IIIa. Cyclic RGD (Arg-Gly-Asp-D-Phe-Cys) is a cyclic conformation of RGD peptides, and has been reported to have much higher affinity and selectivity for binding to the glycoprotein (GP) IIb/IIIa receptor than does its linear counterpart.

In summary, the combination of local low-frequency diagnostic ultrasound radiation and targeted urokinase-containing microbubbles was effective in producing thrombolysis of femoral artery clots in a rabbit model. An ultrasonic frequency of 2.2 MHz and a urokinase dose of 90,000 IU/kg produced optimal thrombolytic effects. By adjusting the ultrasonic parameters, it is possible that the doses of urokinase and radiation ultrasound may be further reduced, thus potentially decreasing treatment side effects.

## Supporting Information

S1 Fig Femoral artery thrombosis without thrombolytic therapy. A mixed thrombus with platelet and fibrin was found in the endovascular lumen, with platelet trabecular formation
(TIF)

S2 Fig A completely dissolved femoral artery thrombus after thrombolytic therapy. The vascular lumen, with completely dissolved thrombus, had unclear thrombus-vascular borders with liquefied platelets and a lumen recanalization rate > 75%. Only a small number of red blood cells and inflammatory cell were found adhered to endothelial cells.
(TIF)

S3 Fig A completely dissolved femoral artery thrombus after thrombolytic therapy. The vascular lumen, with completely dissolved thrombus, had unclear thrombus-vascular borders with liquefied platelets and a lumen recanalization rate > 75%. Only a small number of red blood cells and inflammatory cell were found adhered to endothelial cells.
(TIF)

S4 Fig A partially dissolved femoral artery thrombus after thrombolytic therapy. Mixed thrombus was present in the partially dissolved vascular cavity, with a cavity at the edge or center of the thrombus. Scattered granular platelet trabecula were observed.
(TIF)

S5 Fig A partially dissolved femoral artery thrombus after thrombolytic therapy. Mixed thrombus was present in the partially dissolved vascular cavity, with a cavity at the edge or center of the thrombus. Scattered granular platelet trabecula were observed.
(TIF)

S6 Fig A partially dissolved femoral artery thrombus after thrombolytic therapy. Mixed thrombus was present in the partially dissolved vascular cavity, with a cavity at the edge or center of the thrombus. Scattered granular platelet trabecula were observed.
(TIF)

S7 Fig An undissolved femoral artery thrombus after thrombolytic therapy. The vessels that were not re-canalized had numerous endovascular red cells, scattered inflammatory cells, and mixed thrombi occupying the lumen, and a small portion of dissolved granular platelets were present with lumen recanalization rates of 15%–50%.
(TIF)

S8 Fig An undissolved femoral artery thrombus after thrombolytic therapy. The vessels that were not re-canalized had numerous endovascular red cells, scattered inflammatory cells, and mixed thrombi occupying the lumen, and a small portion of dissolved granular platelets were present with lumen recanalization rates of 15%–50%.
(TIF)
