# Supplementary figures and images for: Combined Low-Frequency Ultrasound and Urokinase-Containing Microbubbles in Treatment of Femoral Artery Thrombosis in a Rabbit Model
Source: PLoS One. 2016 Dec 29;11(12):e0168909. doi: 10.1371/journal.pone.0168909 (PMC5199065; doi:10.1371/journal.pone.0168909)

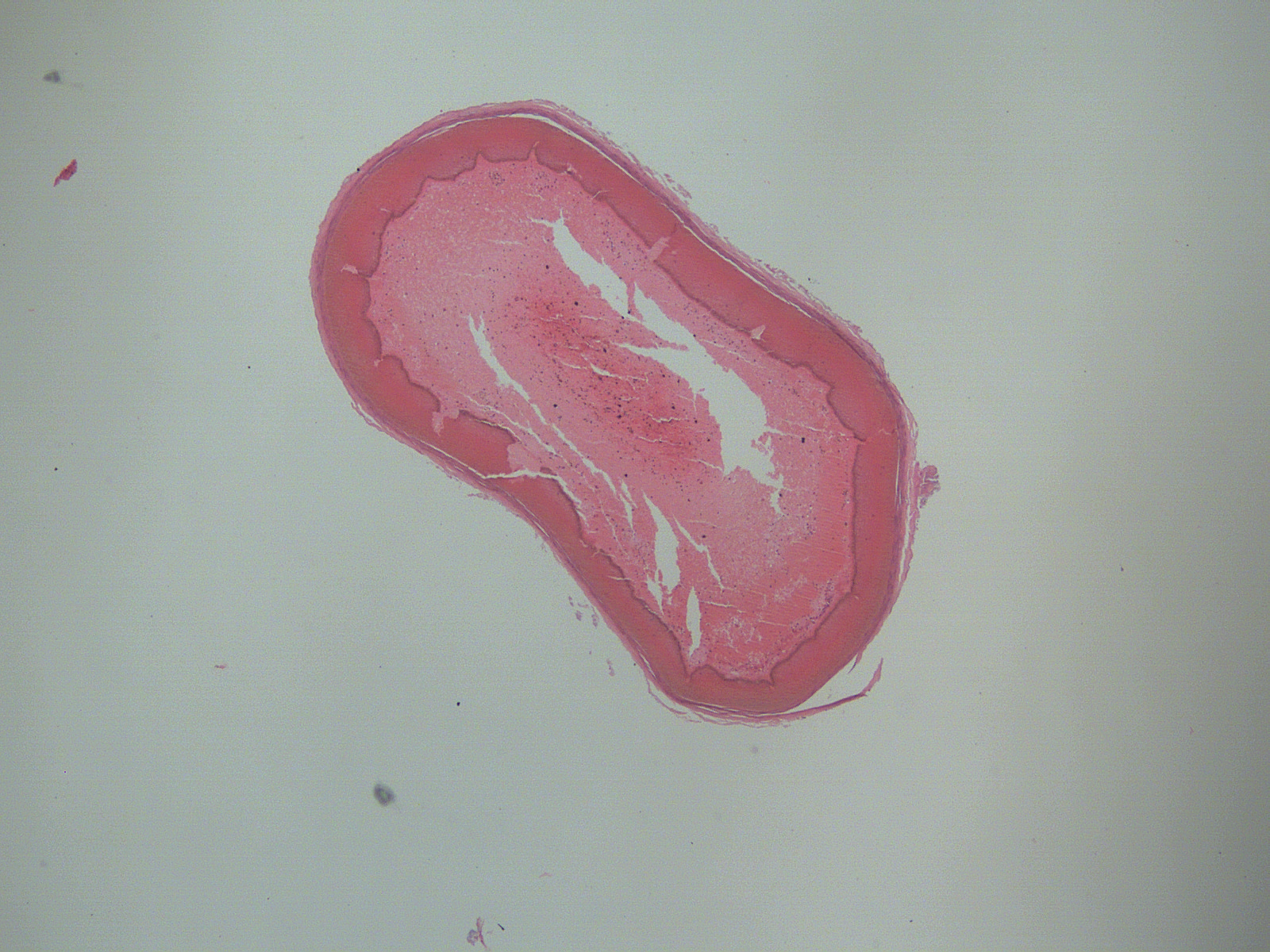

Supplement: S1 Fig — A mixed thrombus with platelet and fibrin was found in the endovascular lumen, with platelet trabecular formation (TIF) [file pone.0168909.s001.tif]

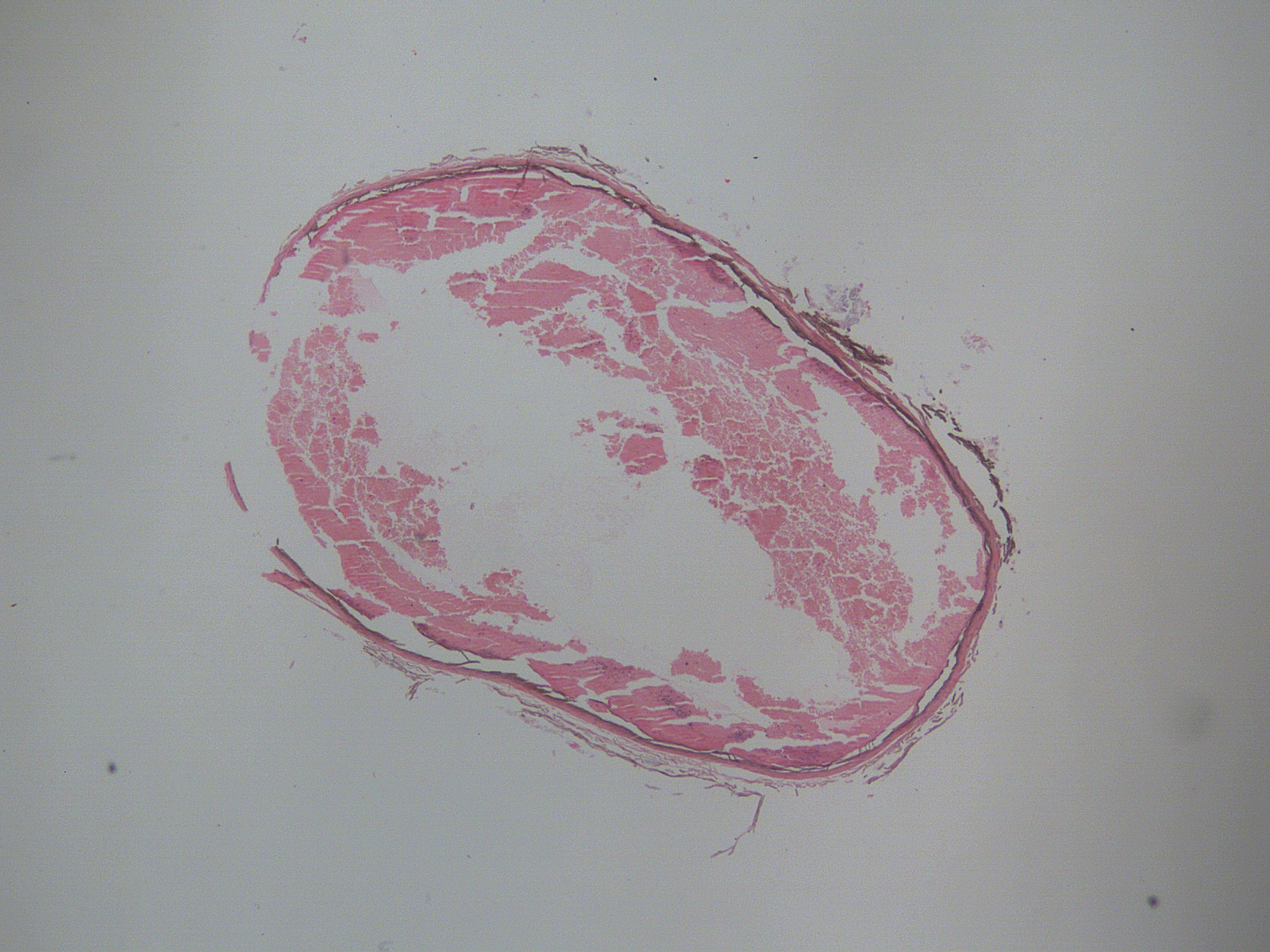

Supplement: S2 Fig — The vascular lumen, with completely dissolved thrombus, had unclear thrombus-vascular borders with liquefied platelets and a lumen recanalization rate > 75%. Only a small number of red blood cells and inflammatory cell were found adhered to endothelial cells. (TIF) [file pone.0168909.s002.tif]

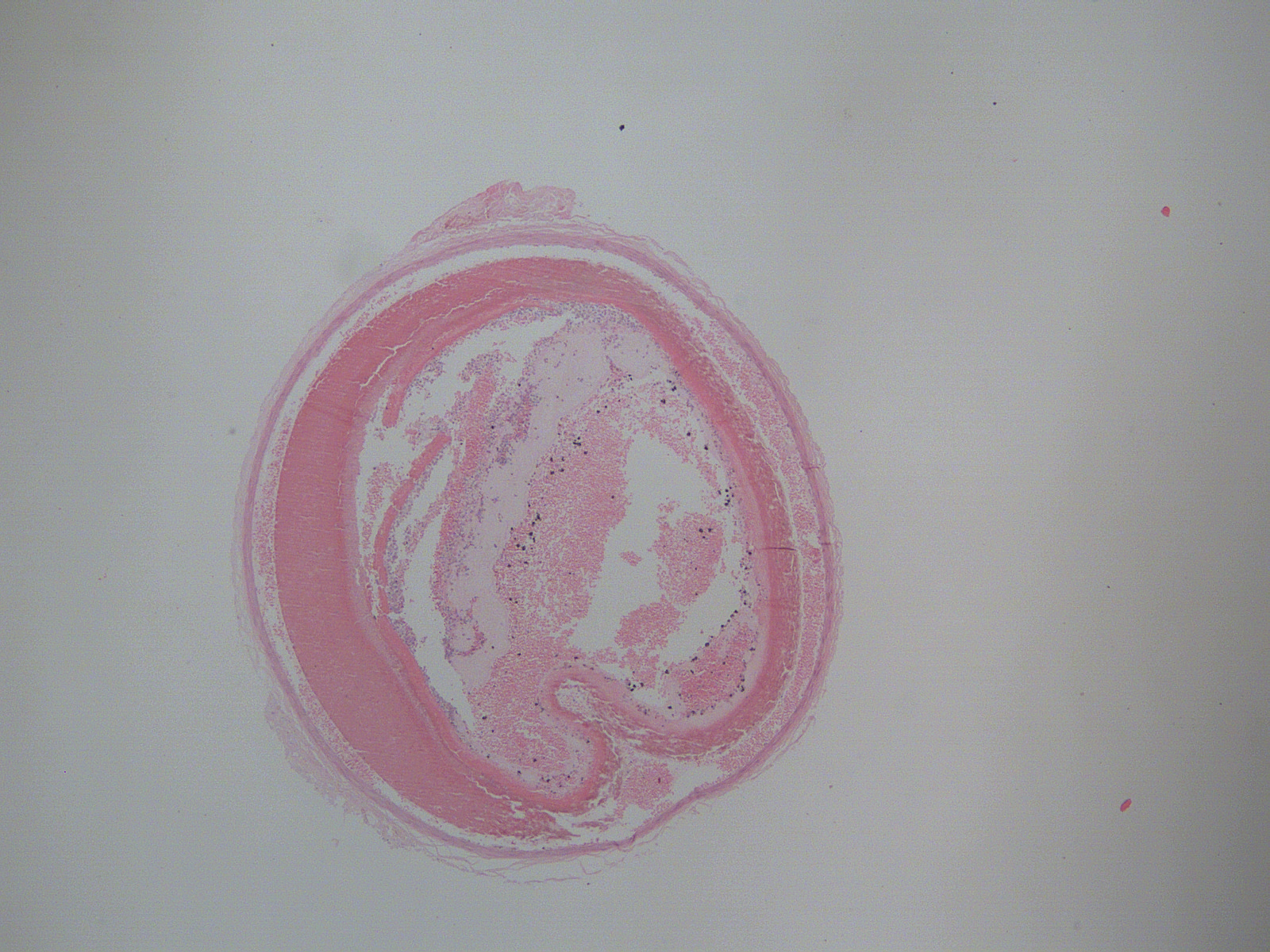

Supplement: S3 Fig — The vascular lumen, with completely dissolved thrombus, had unclear thrombus-vascular borders with liquefied platelets and a lumen recanalization rate > 75%. Only a small number of red blood cells and inflammatory cell were found adhered to endothelial cells. (TIF) [file pone.0168909.s003.tif]

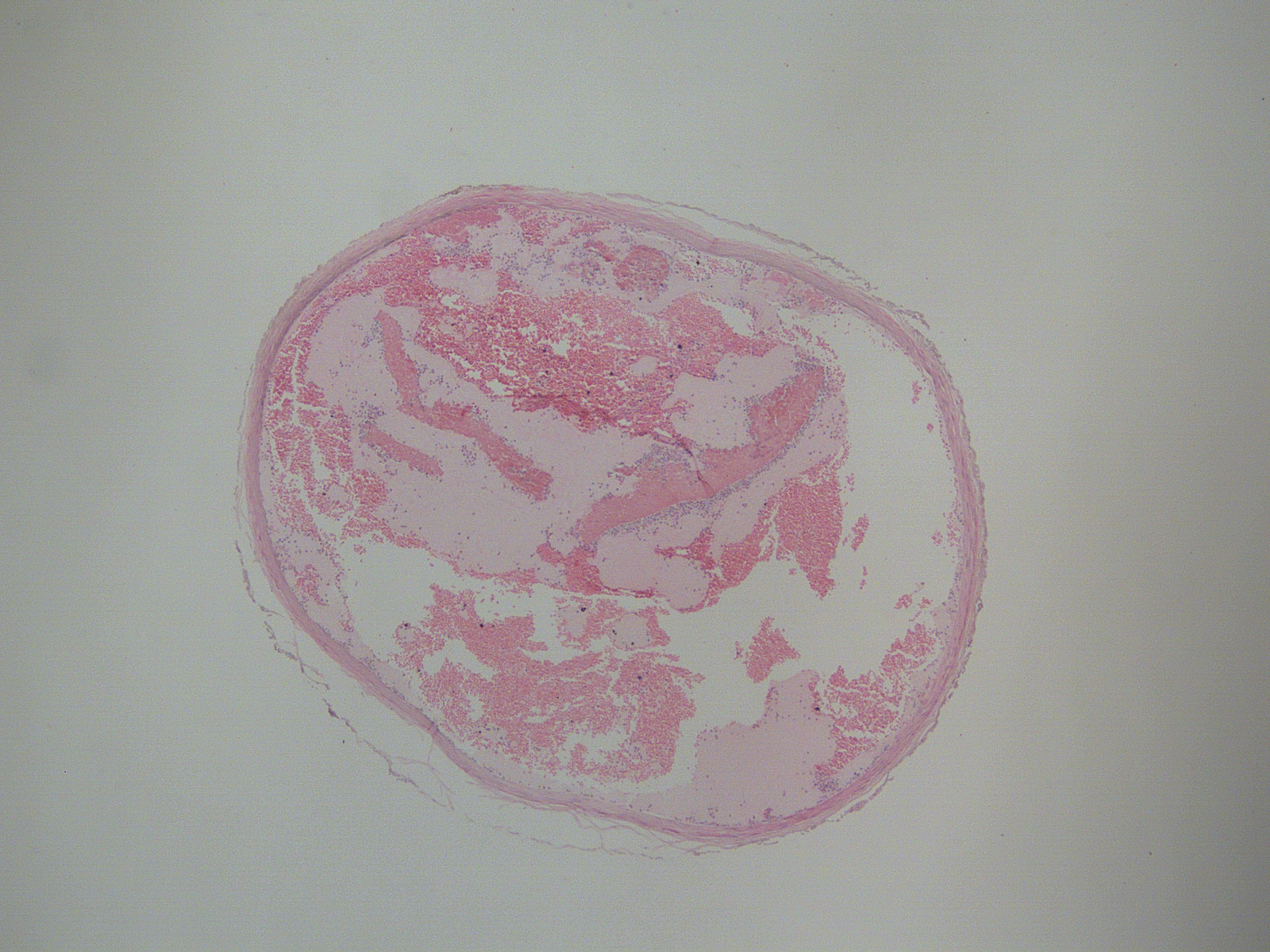

Supplement: S4 Fig — Mixed thrombus was present in the partially dissolved vascular cavity, with a cavity at the edge or center of the thrombus. Scattered granular platelet trabecula were observed. (TIF) [file pone.0168909.s004.tif]

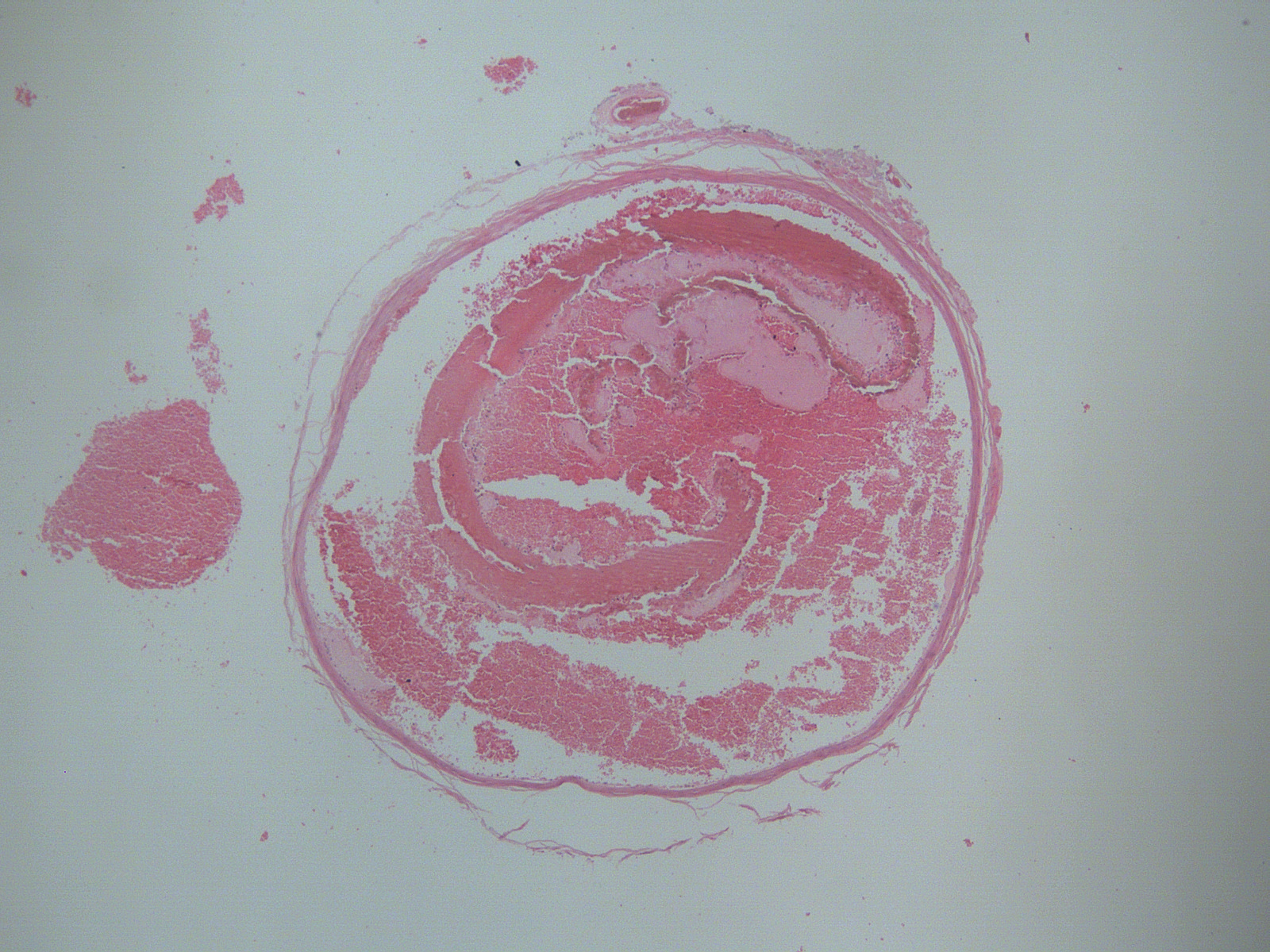

Supplement: S5 Fig — Mixed thrombus was present in the partially dissolved vascular cavity, with a cavity at the edge or center of the thrombus. Scattered granular platelet trabecula were observed. (TIF) [file pone.0168909.s005.tif]

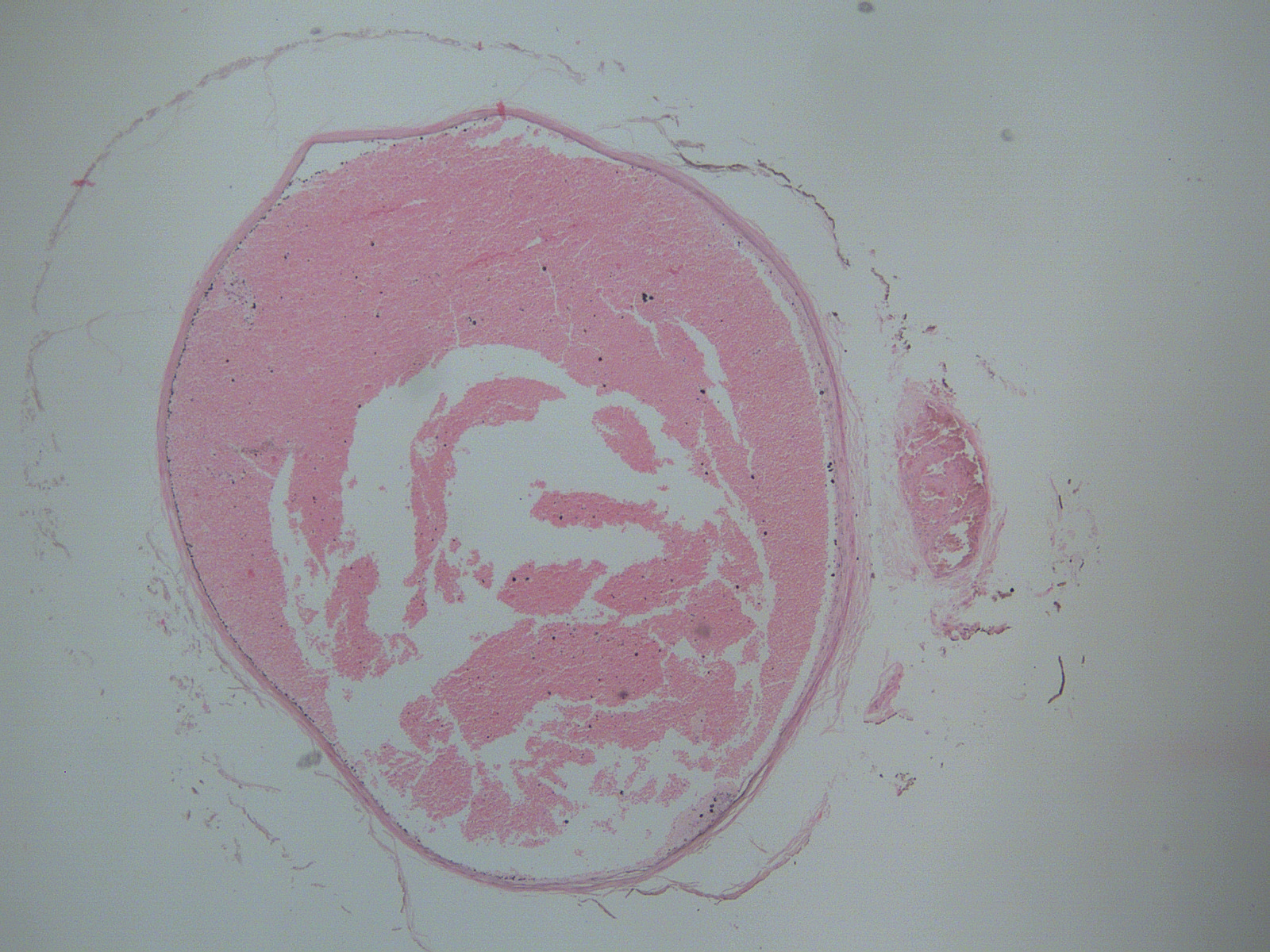

Supplement: S6 Fig — Mixed thrombus was present in the partially dissolved vascular cavity, with a cavity at the edge or center of the thrombus. Scattered granular platelet trabecula were observed. (TIF) [file pone.0168909.s006.tif]

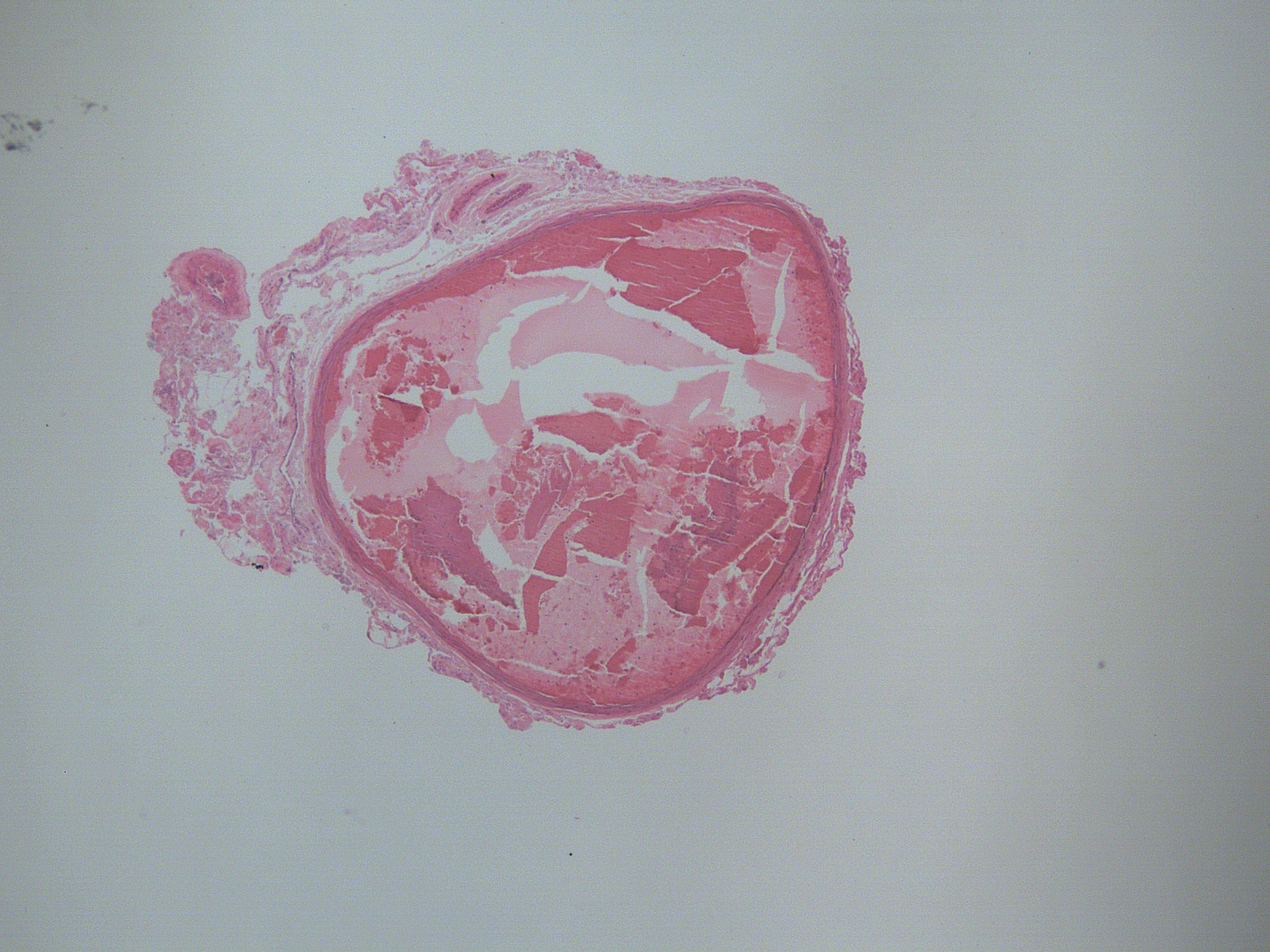

Supplement: S7 Fig — The vessels that were not re-canalized had numerous endovascular red cells, scattered inflammatory cells, and mixed thrombi occupying the lumen, and a small portion of dissolved granular platelets were present with lumen recanalization rates of 15%–50%. (TIF) [file pone.0168909.s007.tif]

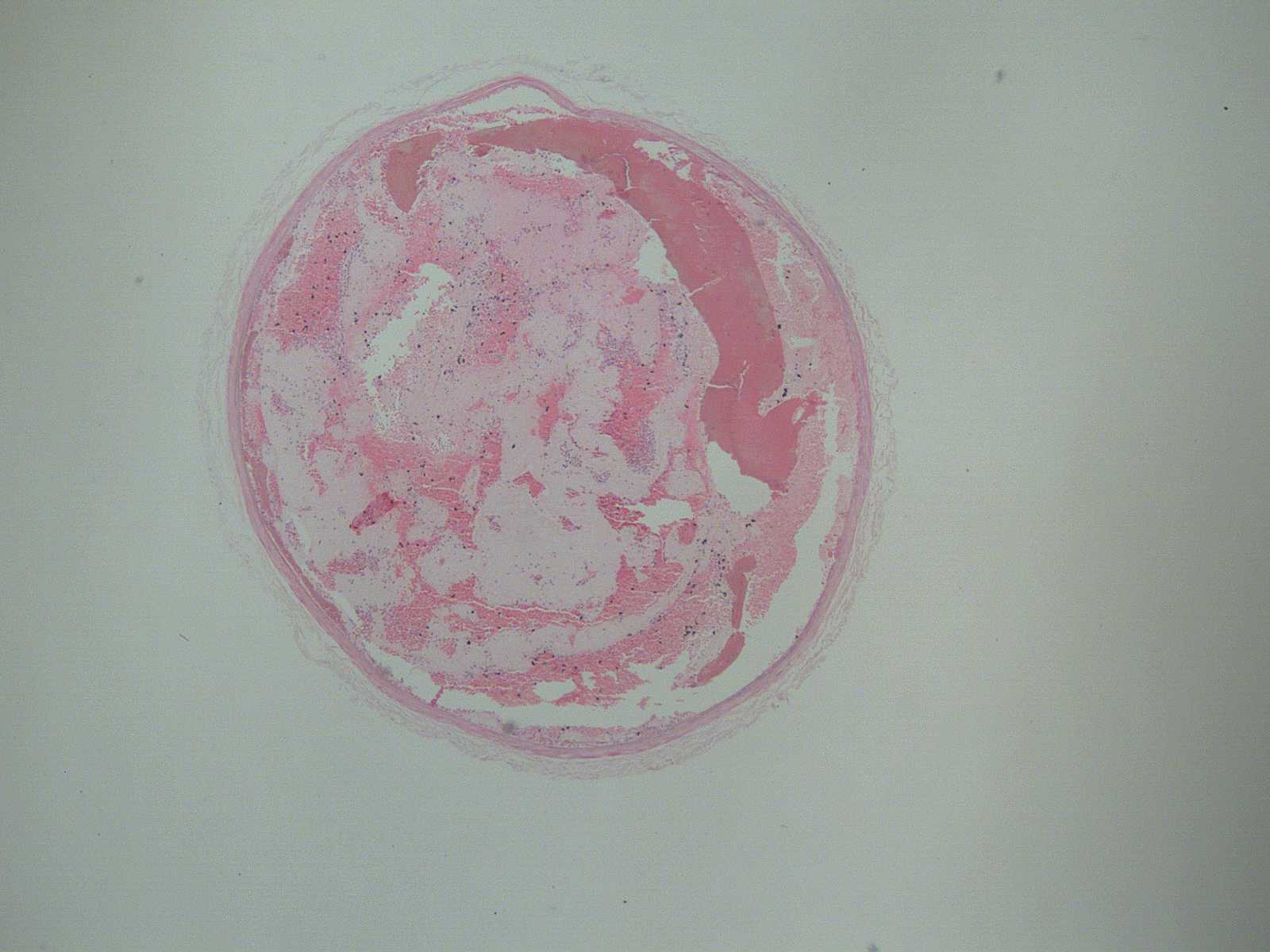

Supplement: S8 Fig — The vessels that were not re-canalized had numerous endovascular red cells, scattered inflammatory cells, and mixed thrombi occupying the lumen, and a small portion of dissolved granular platelets were present with lumen recanalization rates of 15%–50%. (TIF) [file pone.0168909.s008.tif]
